# Characterizing the Heterogeneity of Aging: A Vision for a Staging System for Aging

**DOI:** 10.3389/fpubh.2021.513557

**Published:** 2021-10-12

**Authors:** Efraim Jaul, Jeremy Barron

**Affiliations:** ^1^Geriatric Skilled Nursing Department, Herzog Hospital, Jerusalem, Israel; ^2^Hebrew University of Jerusalem, Jerusalem, Israel; ^3^Division of Geriatric Medicine and Gerontology, Johns Hopkins University, Baltimore, MD, United States; ^4^Chronic Respiratory Department, Herzog Hospital, Jerusalem, Israel

**Keywords:** chronologic age, aging, functional status, anticipatory guidance, life expectancy

## Abstract

**Introduction:** Older adulthood encompasses several decades of change and heterogeneity. Primary care providers need a geriatric comprehensive vision for defining older adult subpopulations.

**Methods:** Using PubMed and Google searches, we reviewed the literature on epidemiology of age-related physiological changes, age-related diseases and geriatric syndromes, functional state, and emotional and social changes. We divided old age into strata based on chronological age and strata based on functional state, disease burden, and geriatric syndromes.

**Results:** We describe 4 chronological-age strata beginning at age 60, and 4 functional-age strata based on frailty according to a modified clinical frailty scale. We provide clinical considerations and anticipatory guidance topics for each of the age strata and functional strata.

**Conclusion:** Chronological age, functional status, chronic disease burden and geriatric syndromes, and life expectancy are all important domains that impact clinical care and appropriate anticipatory guidance for individual older adults. Better knowledge for differentiating subpopulations of older adults may improve clinical care, reduce medical overuse, improve personalized anticipatory guidance, and focus on the impact of functional state on the quality of life.

## Introduction

In the coming decades, the proportion of older adults in the world will nearly double (1). Older adults are a heterogeneous population, with many people over the age of 80 continuing to work and travel, while others might be weak, chronically ill, or disabled. A traditional framework for describing different populations of older adults is “young-old,” “old,” “old-old,” and/or “oldest old” (2). Fried's frailty phenotype (3) is a similar three-stage framework in which people are classified as non-frail, prefrail, or frail. Proteomic analysis finds large changes in gene expression at about the age of 40, 60, and 80 (4). However, these frameworks are not adequate to describe the different stages of aging and subpopulations of older adults. Older age is a risk factor not only for normative physiological changes with aging but also for cancer, heart disease, diabetes, dementia, and many other chronic conditions. Genes, disease, and behaviors can pull on the chronological age of a person and make the person appear younger or older with the risk profile (“biological age”) of someone younger or older (5).

The changes associated with aging can be divided into a few domains. First, there are normative physiological changes in every organ system as a person ages. Second, many diseases become more prevalent in older age. These physiological changes and diseases can contribute to functional limitations or disability. Third, aging is associated with some emotional and psychological changes. Finally, many social and environmental changes are common with advancing age.

Although adults age in different patterns and with different trajectories, generalizations about normal and typical aging might be helpful to inform patients, clinicians, and policymakers. When caring for older adults as a clinician or as a family caregiver, predicting the future and then planning for the most likely aging trajectories are the key steps. A primary care provider needs to consider life expectancy and time to benefit before ordering screening tests and before prescribing certain medications. Although many developed nations have an increasingly aging population, health care providers often display inadequate knowledge of geriatric medicine and low confidence in their ability to care for complex multimorbid older patients (6). Primary care providers and other physicians want to know when and how to treat older patients differently based on their age, comorbidities, and other factors (7). Primary care providers should be able to counsel older patients and their families on what to expect in the future and how to plan. Meanwhile, family caregivers who are helping to identify an appropriate placement (even at home) for older adults who are unable to live independently need to anticipate their future functional state and needs (8). Moreover, policymakers considering how to prepare for the “silver tsunami” (1) must appreciate the needs of different older adult subgroups.

At every stage during childhood, anticipatory guidance (9, 10) and a review for parents on what to expect and what to worry about it in the future, including evidence-based strategies for prevention, screening, counseling, and referrals were provided by the health care team. In contrast, in older age, which now spans 30 years rather than a few years, there is typically no anticipatory guidance plan to deal with age and its results.

This study presents a model for the division of older age into stages, which are characterized by chronological age, functional status, chronic disease burden, and geriatric syndromes. Dividing aging into stages and subpopulations can have two discrete benefits, such as improving the delivery of appropriate anticipatory guidance to patients and families, and improving clinical care by targeting interventions appropriately and avoiding inappropriate care [medical overuse (11)].

## Methods

The study began with a literature review using PubMed and Google. Literature reviews included search terms such as “age-related diseases, “normal aging,” “physiological aging,” “emotional changes with aging,” “social changes with aging,” “functional changes with aging,” and for specific conditions or changes that were identified, the terms dementia, hyperlipidemia, and loneliness were paired with “epidemiology” and “aging.” We then divided older age into four reasonable chronological age strata. For each chronological age strata, we described normative changes, age-related diseases, emotional, and social changes. Many of these changes are gradually progressive, and the designated age strata reflect an acceleration of change or the peak of a curve. Clinical considerations for the health care team based on these findings are suggested. We also divided aging into four functional strata and explored clinical considerations and anticipatory guidance topics that might be appropriate for each stratum. Tables were reviewed iteratively.

The stages of chronological aging in this study begin at age 60, but in fact, some body systems reach their peak performance well-before age 60. Age 60–69 was chosen because many physiological changes become clinically evident, and social and psychological changes are also noteworthy at this age.

We proposed an aging staging system using chronological age, functional status/disease burden, and life expectancy as three separate domains, comparable with the TNM oncology staging system (12) (tumor size, lymph nodes, and metastasis).

Functional status categories are based on compression of the Rockwood Clinical Frailty Scale (13). Life expectancy can be expressed as an expected age at death, or as the number of predicted additional years of life, or as the number of additional disability-free years (14), or as mortality risk (15) (1, 5, and 10 years). Life expectancy can be predicted based on age, functional status, disease burden, and geriatric syndromes.

## Results

Chronological age stages can be helpful in clinical care, particularly related to physiological and emotional changes, and prevalence of disease may vary with age. The four stages based on age (see Table 1) begin at age 60–69.

**Table 1:** Chronological age strata with appropriate clinical considerations and anticipatory guidance topics.

**Stage 1 age 60–69**	**Clinical considerations**
**Normal aging**	
	Aerobic exercise maintains fitness
	Avoid dehydration and nephrotoxins
	Social engagement and mentally challenging activities may help preserve cognition
	Reading glasses often needed (16) Night driving may be more challenging
	Counsel on menopausal treatment (17) Ask about sexual health
	Resistance exercise is helpful to minimize muscle loss
**Disease**	
	Check blood pressure at clinic visits (18)
	Lifestyle management and consider pharmacotherapy for hyperlipidemia according to guidelines
	Avoid anticholinergic medication to minimize burdensome adverse effects
**Emotional**	
	Older adults may not complain of symptoms that affect quality of life (19)
**Social**	
	Retirement planning
	**Anticipatory guidance topics**
	•Cardiac risk factor modification for men •Vision changes •Cancer screening
**Stage 2 age 70–79**	**Clinical considerations**
**Normal aging**	
	Hearing screening, counseling (20, 21), and referral
	Counseling on oral health (22), avoid anti-cholinergic medication
	Fast decline or very slow walking speed predicts mortality (23)
	Vaccines less effective (24)-consider HD Flu vaccine
**Disease**	
	Cardiac Risk factor management
	•Weight loss and exercise is helpful to reduce arthritis symptoms •Avoid non-selective NSAIDS for arthritis
**Emotional changes**	
	Opportunity for mentoring others in healthy behaviors (25)
**Social changes**	
	Counseling: goals, learning, spirituality, volunteerism
	**Anticipatory guidance topics**
	•Hearing assessment •Dental health •Cardiac risk factor modification for men and women •Changes in daily activities –work, volunteering •Vaccines for older adults •Bone health for women
**Stage 3 age 80–84**	**Clinical considerations**
**Normal aging**	
	Social engagement, physical activity, and cognitive stimulation are likely protective of cognitive decline
	Consider pharmacokinetics when prescribing
	Do not over treat abnormal urine cultures
**Disease and geriatric syndromes**	
	Bone density screening after age 65 for women Vitamin D screening Treatment/supplementation
	•BMI screening and weight management •Glucose screening for those at high risk
	•Falls Risk screening •Physical activity and multifactorial interventions
	•Fall prevention •Home safety screening by occupational therapist
	Screening and referral for assistive listening devices
	•Estimate life expectancy •Explain risks and benefits of screening tests
	Vision Evaluation and referral
	Ask about urinary incontinence. Counseling and referral
	For dementia patients: •Counsel on cognitive + neuropsychiatric symptoms •Maintain social and cognitive stimulation •Financial and legal planning
**Emotional changes**	
	Decision aids, particularly related to numeracy. Avoid offering too many choices (26)
	Depression screening and treatment
**Social changes**	
	PT/OT evaluation for identified mobility limitations (27)
	Encourage social engagement and physical activity
	**Anticipatory guidance topics**
	•Maintaining cognitive health •Cardiac risk factor modification for men and women •Joint health •Fall prevention •Pain •Depression •Financial exploitation
**Stage 4 age 85+**	**Clinical considerations**
**Normal aging**	
	Gait and nutritional evaluations
**Disease and geriatric syndromes**	
	Prevent iatrogenesis Optimize transitional care
**Emotional changes**	
	Screening and referral if suicide risk is identified (28)
**Social changes**	
	Consider risk of social isolation
	Care planning with patients and families
	**Anticipatory guidance topics**
	•Polypharmacy •Functional changes and functional assessment •Nutrition and nutritional Assessment •Dangers of social Isolation

During this period, pulmonary (29) and renal (30) function, as well as some neurological (31) functions, start gradually declining, usually without clinical signs. Atherosclerosis (32) in men often progresses. During the second age stage, 70–79, hearing loss (20) becomes more common as well as osteoarthritis (33) and cardiac events (34) in men. Many working people transition toward retirement. Many women are caregivers (35) for ill husbands. During the third age stage, 80–84, cognitive aging (36) is more noticeable, and rate of falls (37) increases. The value of cancer screening (38) becomes more ambiguous due to lag time to benefit, detection of insignificant tumors, and burden of false-positive results. During the fourth stage of aging, 85+, most people are still in good health but problems such as dementia (39) and osteoporotic fractures (40) are increasingly common. An increasing majority of older adults are women and nearly half of these women live alone (41), many with risks for social isolation. Cardiac events in women increase to approximate the rate of men (42). Dementia and disability rates rise with advancing age.

The functional stage system (see Table 2) has four strata beginning with independent and lacking chronic illness and progressing to dependency and disability. The staging system is similar to the ECOG performance status grading system used in oncology (43), but tilted to a more robust population.

**Table 2:** Functional age stages with appropriate clinical considerations and suggested anticipatory guidance topics.

	**Clinical considerations**	**Anticipatory guidance topics**
Stage A Independence and minimal chronic illness burden (CFS 1-3)	1. Counsel on compliance with medication and lifestyle plan. 2. Regular medical visits and perhaps telehealth disease management for those with certain chronic illnesses.	1. Travel safety 2. Group physical activity opportunities 3. Work and Volunteer opportunities 4. Hot weather safety 5. Driving safety
Stage B Independent with some impairments (pain, balance, slower, etc.) due to worsening chronic illnesses (CFS 4)	Minimize polypharmacy and drug-disease interactions	1. Fall prevention 2. Avoiding polypharmacy 3. Financial planning for possible need for long term care 4. First discussions of assistive technologies 5. How to get the most out of medical visits
Stage C Mainly independent but some functional limitations and need for help (CFS 5)	1. Avoid medications that could increase risk of falls or cognitive impairment 2. High burden treatments are practical only with intensive social support.	1. Strategies for preventing hospitalizations such as medication compliance and good communication with primary care team 2. Advance directives 3. Home modifications, assistive technologies, and/or discussions of placement 4. Preventing social isolation
Stage D Disability and dependence on support of caregivers (CFS 6-9)	Cancer screening, intensive chemotherapy, and dialysis are probably inappropriate	1. Reassessment of advance directives and end of life care wishes 2. Caregiving plan 3. Elder abuse

In the first functional stage (A) of this model, corresponding to stages 1–3 of the Clinical Frailty Scale, older adults are active, independent, and without heavy burdens of chronic illness. They may take medicines regularly. Counseling during this stage will focus on the prevention of diseases (particularly cardiovascular), healthy lifestyle promotion, continuing work, and social engagement.

In the second functional stage (B), corresponding to Clinical Frailty Scale (CFS) stage 4, the old accumulate more chronic illnesses and display some functional impairments, but they still remain active and independent. They may be interested in technology device options to optimize independence. They may be considering a reduction in their driving.

In the third functional stage (C), corresponding to CFS stage 5, older adults display chronic illnesses of worsening severity, contributing to hospitalizations and impairments such as pain and fatigue. Polypharmacy and falls also begin to cause a decline in functional state and quality of life. People perform less physical activity. They may be interested in home modifications to ensure safety and promote independence. Some people might move to independent-living communities. As this stage progresses, many people become frail and may use assistive devices for mobility. Hospitalizations become more frequent and older adults may need more supervision from family caregivers. Mild cognitive impairment or mild dementia also can occur in this stage. Some people will move to assisted living communities.

In the fourth functional stage (E), corresponding to CFS 6–9, older adults need regular caregiving help due to disability (physical or cognitive). Without adequate support from caregivers, older adults may need placement in nursing care facilities. Support for caregivers is important. As this stage progresses, the responsibilities of caregivers grow and life expectancy becomes short. Hospitalizations are burdensome, and benefit from treatment is hard to predict.

### Examples

Patient: M is a 73-year-old married part-time cashier with chronic obstructive pulmonary disease (COPD) and hypertension. She has not had any hospitalizations in the past year but has had several primary care and specialist visits as well as urgent care visits. She takes six medicines including her inhalers. She is not limited by shortness of breath with her daily activities.

#### Age Stage: 2A, 10% Risk of 5-Year-Mortality

Patient: S is an 87-year-old retired dentist with moderate dementia as well as coronary artery disease, congestive heart failure, knee osteoarthritis, and benign prostatic hypertrophy, who lives in the memory care section of an assisted-living facility. His wife visits daily. He was hospitalized this year once for a CHF exacerbation and once for complicated urinary tract infection (UTI). He takes eight medications regularly. He walks with a walker or with the assistance of another person.

#### Age Stage: 4D, Life Expectancy 3 Years

Anticipatory guidance based on age (see Table 1) reflects common age-related physiological and emotional/social changes. Anticipatory guidance based on functional state and burden of chronic illnesses (see Table 2) will include health promotion as well as the limitations that one might expect with a personal profile of chronic conditions, and might also include planning for future housing needs, assistive technologies, and caregiver support.

## Discussion

Aging can be viewed as a very slow step-wise decline from wellness and independence toward disability, reduced quality of life, and ultimately death. The rhythm of decline of an individual is very personal and depends on the genes, lifestyle, diseases, and geriatric syndromes such as dementia and falls. Social support and psychological factors like resilience and optimism also have a relevant effect. Not all age-related changes reflect functional decline and incident disease. In fact, migraine headaches and allergic rhinitis symptoms tend to decline with age. Meanwhile, wisdom and crystallized intelligence increase with age, and emotional situations are appraised more positively.

The biological causes for physiological changes with aging and for the prevalence of age-associated diseases continue to be explored and debated. Theories include accumulation of reactive oxygen species, telomere shortening, proinflammatory cytokines, apoptosis, and impaired repair systems (44).

Dividing the process of aging into phases, and characterization of these phases from various aspects can raise awareness and recognition that old age is not homogeneous or stereotypical as it is often considered. Healthy, active people in their 70's should not be treated like disabled people in their 90's, and active people in their 90's should not be treated like sick, disabled people in their 90's. Awareness of age-related physiological changes (such as reduced acuity of vision and hearing, slower reaction time, and impaired balance) will prepare patients and caregivers to prevent trauma, falls, and medication adverse effects.

Cumulative reports in the medical literature of longitudinal studies in older populations describe normative physiological signs/markers related to aging, which are predictive of future morbidity and mortality (gait speed, frailty, renal function, cardiorespiratory fitness, etc.). Recognition of these biological physiological markers helps to risk-stratify the older population with goals of slowing, delaying, and even preventing age-related changes.

Physiological changes in an older person occur not only from biological–physical causes, but also from social, emotional, and mental health circumstances. Awareness of these circumstances may improve or even prevent age-related deterioration, such as awareness and attention to depression and suicide in men during the 1st year after the loss of a spouse.

Adults in stages one and two of aging (age 60–79) typically remain in the first stage of the four-phase functional scale. The rate of decline to the next phases depends on vascular risk factors, genetics (family history), and social/environmental factors (such as education, career, physical activity, and social engagement or isolation). The goal of medicine is to compress morbidity, allowing old and old-old adults to spend more time in these earlier functional aging phases and less time in the disabled or burdened phase with bothersome symptoms.

Although telomere length and tools to calculate biological age have benefits over chronological age, they have not yet been adopted into clinical practice. Frailty scales are not sufficient for use in many settings, such as for the U.S. Preventive Services Task Force (USPSTF) recommendations. These scales and tools have particular uses in particular settings, and there is a risk of using them inappropriately (45). There are settings in which chronological age is appropriate to consider and settings in which frailty and life expectancy are appropriate. The settings in which biological age or telomere length are helpful need further clarification. The health care team should mainly consider the stage of the chronological age of a person when trying to make decisions about vaccines and low-burden screening tests. The health care team should mainly consider the stage of functional age of a person when trying to make decisions about home safety and about burdensome treatments like chemotherapy or hemodialysis. Finally, the health care team should consider the life expectancy of a person when making decisions about tests and treatments with a long lag time to benefit (46). Online tools like http://eprognosis.ucsf.edu can facilitate estimation of life expectancy.

Chronological age stratifications may not correlate with medical, functional, emotional, and social changes that an individual may be experiencing for multiple reasons. The chronological age strata used in this study are obviously inexact, but useful. As noted, older adults are heterogeneous and may develop problems earlier than average or later or never. The final chronological age strata in this proposed staging system are >85. In fact, there may be substantial differences between 87-year-olds and centenarians. However, at this time, there is insufficient data to describe organ system changes and disease epidemiology in subpopulations like centenarians.

Anticipatory guidance in Tables 1, 2 can help patients and families recognize and manage age-related changes. However, the most effective behavioral and pharmacological prevention strategies may need to begin decades before the advent of these adverse conditions. For example, preventing cardiovascular events or dementia may require management or avoidance of atherosclerotic risk factors starting in young adulthood.

With the advancement of modern medicine and with improved preventive care and lower rates of infectious disease, we more often witness an expanding gap between chronological age and biological age. Some people with advanced age have functional and cognitive abilities that are greater than those of younger people.

Just as the term “successful aging” suggests that some people are aging successfully and some are not, a staging system for aging may seem unkind and pejorative to people in the final stages. Many view the use of chronological age as a source of ageism. A large proportion of older adults experience ageism in the workplace and in their daily lives. The entire community has a responsibility to replace negative stereotypes of aging positive and nuanced portrayals. In fact, there is meaning and opportunity in every stage of life including every stage of aging. As people age, they continue to have a desire to be generative, which can include work, volunteering, grandparenting, and storytelling. Even people with very advanced illnesses can have dignity and be a source of inspiration as well as a motivator for kind acts.

Despite differences among individuals, aging can be divided into strata by chronological age, functional age, and disease burden, and by short or long life expectancy. These strata can be helpful to practitioners for the purpose of providing the required anticipatory guidance and high-quality care. Chronological age itself is important to help predict risk for physiological and pathophysiological changes in organ function, but also stratifying by functional state and by life expectancy enable the health provider to provide guidance based on risk for future functional decline, health care utilization, and the likelihood of benefitting from particular tests and treatments.

It would be useful to describe the aging stages of older adults in a large observational study and describe their future functional and health outcomes. It would also be useful to explore the willingness of primary care providers to use a staging system to stratify their older patients.

## Data Availability Statement

The original contributions generated for the study are included in the article/supplementary material, further inquiries can be directed to the corresponding author/s.

## Author Contributions

All authors listed have made a substantial, direct and intellectual contribution to the work, and approved it for publication.

## Conflict of Interest

The authors declare that the research was conducted in the absence of any commercial or financial relationships that could be construed as a potential conflict of interest.

## Publisher's Note

All claims expressed in this article are solely those of the authors and do not necessarily represent those of their affiliated organizations, or those of the publisher, the editors and the reviewers. Any product that may be evaluated in this article, or claim that may be made by its manufacturer, is not guaranteed or endorsed by the publisher.
